# What’s the point? The functional role of claws in pad-bearing taxa (Gekkota: Diplodactylidae)

**DOI:** 10.1098/rspb.2025.1362

**Published:** 2025-09-03

**Authors:** Rishab Rajan Pillai, Jendrian Riedel, Wytamma Wirth, Slade Allen-Ankins, Eric Nordberg, Will Edwards, Lin Schwarzkopf

**Affiliations:** ^1^ College of Science and Engineering, James Cook University, Townsville, Queensland, Australia; ^2^ School of Biological Sciences, The University of Adelaide, Adelaide, South Australia, Australia; ^3^ Department of Evolutionary Biology, Bielefeld University, Bielefeld, Germany; ^4^ Leibniz Institute for the Analysis of Biodiversity Change, Bonn, Germany; ^5^ Core Facility for Multidisciplinary Structural Analysis & Biological Structures and Biomimetics, Hochschule Bremen, Bremen, Germany; ^6^ College of Public Health, Medical and Veterinary Sciences, James Cook University, Townsville, Queensland, Australia; ^7^ The Peter Doherty Institute for Infection and Immunity, Melbourne, Victoria, Australia; ^8^ School of Environmental and Rural Science, University of New England, Armidale, New South Wales, Australia; ^9^ College of Science and Engineering, James Cook University, Cairns Campus, Cairns, Queensland, Australia

**Keywords:** *Oedura*, *Strophurus*, attachment, shear force, substrates, clinging

## Abstract

Morphological adaptations facilitate effective movement within habitats. Claws are among the most common adaptations enabling organisms to use inclined and vertical surfaces. However, some taxa have evolved adhesive pads in addition to claws, with claws suggested to be more effective at gripping coarse surfaces, while pads attach better to fine-grained surfaces. Using test surfaces that represented the range of surface roughness used by six species of diplodactylid geckos in nature, we quantified the role of claws and pads acting together, and of pads alone. We examined two functional traits, attachment (on inclines, 45° and vertical surfaces, 90°) and clinging ability (on inclines only). Claws were critical to attachment on vertical surfaces, and attachment declined linearly with decreasing surface roughness. Although attachment was lowest on fine-grained surfaces, this was where claws had the greatest functional contribution. Clinging ability also declined linearly with decreasing surface roughness, where claws played an additive role. Our study highlights novel results describing the function of gecko adhesive systems on different surfaces and suggests a clade-specific interaction of claws and pads. Specifically, we highlight that pads alone can be capable of attachment on rough surfaces, with claws contributing more on fine-grained surfaces.

## 1. Introduction

Morphological adaptations facilitate locomotion over various microhabitats and surfaces [1,2]. Claws, which are widespread in vertebrates and invertebrates, aid movement by providing grip [3]. Many taxa have, however, evolved adhesive pads in conjunction with claws, also enhancing attachment [4–6]. Why have two different methods of attachment? Presumably, the context-dependent function of each component influences the type and diversity of surfaces that can be effectively traversed [7,8]. Presently, the function of claws and pads within the same system has primarily been studied in invertebrates [9–16] and a few lizards [6,17–20], suggesting that claws perform well on coarse surfaces by interlocking with large asperities that are less accessible to adhesive pads [21–25], whereas pads are more effective on smoother surfaces [7,21,26–29], adhering when a high surface area is available [30,31]. The independent and combined roles of these structures potentially determine the range of microhabitats an organism can use. Investigating the relative roles of claws and adhesive pads may help clarify their function in different organisms.

Among lizards, geckos are the most diverse and speciose pad-bearing clade, exhibiting wide variation in the morphology of the claws (including partial or complete claw reduction in some pad-bearing species) [32,33] and especially extensive variation in pad morphology [34,35], suggestive of potential clade-specific fine-scale differences in the interaction of surfaces with claws and pads. Despite this diversity, studies on morphology and performance in geckos have primarily focused on selected species in the family Gekkonidae with ‘basal’ toepad morphology and relatively large claws. These studies report a linear decline in clinging performance as surface roughness decreases [18,30], consistent with the above-stated idea that claws are used for attachment to rough surfaces, and pads to smoother surfaces.

Studies examining clinging in geckos have used a range of surfaces, although they have chiefly been artificial, with an emphasis on ensuring experimental conditions were precisely regulated and standardized [31,36–39]. In nature, geckos use a wide range of plant and rock surfaces with highly diverse surface properties [38,40]. For meaningful evolutionary and ecological interpretations, information on performance on these natural surfaces would be needed [38]; however, natural substrates are difficult to control for comparisons of clinging ability. A middle road is to control surface type, while allowing an important variable (e.g. roughness) to vary in a way representative of surfaces in nature [17]. This approach is important to increase the ecological relevance of experimental measures; thus, we have used this approach to provide an insight into the responses of claws and adhesive pads to surfaces with similar roughness to those used by diplodactylids in nature.

Australian diplodactylid geckos are evolutionarily distinct [41], significantly differing from gekkonids in the morphology of their adhesive apparatus [42]. Unlike most gekkonids, they possess ‘leaf-like’ terminal toepads with small claws [42]. They are equally able to cling to rough and very smooth surfaces, but struggle with fine-grained surfaces [39], suggesting that the simple dichotomy that claws are useful on rough surfaces and adhesive pads for smoother surfaces might not be sufficient to explain performance in this lineage. Thus, diplodactylid geckos provide an interesting alternative system in which to examine the interaction of claws and microfibrillar pads for clinging in vertebrates and an opportunity to explore functional consequences of different pad and claw morphologies among lizards.

We measured ‘attachment’ (the probability of adhering to a substrate versus falling off) and ‘clinging ability’ (shear force generated on inclines) as separate variables on artificial surfaces that directly correspond to the range of surface roughness encountered by these geckos in nature. First, we investigated whether the small claws typical of many diplodactylid geckos serve a functional role, as this group is characterized by well-developed toepads. We then examined whether there was a trade-off between the effectiveness of claws and toepads on different surfaces, at different orientations or whether they functioned synergistically. If diplodactylid geckos are similar to gekkonids in this regard, we expect their attachment and clinging ability to be highest on coarse surfaces when their claws are intact, as the claws enhance grip when there is little surface area for the pads to adhere to. Conversely, claws would contribute least to attachment on fine surfaces, where the pads maximize contact and thus provide most of the clinging ability.

## 2. Material and methods

### (a) Study species and microhabitat use

We collected six diplodactylid gecko species from different microhabitats to examine whether the function of claws and pads varied based on their ecology from five locations in Queensland, Australia. Geckos were detected using spotlighting surveys and collected by hand. *Oedura castelnaui* (*n* = 9) was found exclusively in arboreal microhabitats, while *O. coggeri* (*n* = 8) was found only in saxicolous microhabitats. *O. cincta* (*n* = 10) and *O. monilis* (*n* = 11) were scansorial generalists, occupying trees, rocks or fallen logs. *Strophurus krisalys* (*n* = 6) and *S. ciliaris* (*n* = 5) were collected from shrubs defined as plants that had multiple stems arising close to the ground.

### (b) Housing

After collection, geckos were housed individually in plastic enclosures (30 × 15 × 9 cm) in temperature-controlled rooms (25°C ± 1.5°C) at the university campus, with a 12 h light and dark cycle (06.00–18.00 L; 18.00–06.00 D). Each enclosure included a ceramic tile shelter, paper towel flooring to protect setal fields and ad libitum water access. To enable thermoregulation, enclosures were placed on racks with heat sources under one end, creating a thermal gradient reaching 33°C during the day. Geckos were fed cockroaches (*Nauphoeta cinerea*) dusted with vitamin and calcium powder (Reptivite™) twice weekly.

### (c) Ecological relevance of test substrates

Our understanding of how claws and adhesive pads function comes from studies that used experimental surfaces well-suited to distinguish the role of each component [19,21,23,43–45]. Although these studies form the foundation of our understanding of how each component interacts with substrates, relatively few studies use surfaces that represent characteristics of surfaces in nature [17,22,24,31,46]. However, diplodactylid geckos use a structurally and chemically diverse range of surfaces, and several studies have suggested the importance of using ecologically relevant substrates [38,47]. To ensure our experimental surfaces were controlled, i.e. the structural and chemical composition of surfaces were similar, but also reflected the roughness of natural substrates, we used fine-scale habitat data, surface roughness measured as peak-to-valley heights (µm), to select appropriate sandpaper coarseness for clinging ability tests.

**Figure 1.**
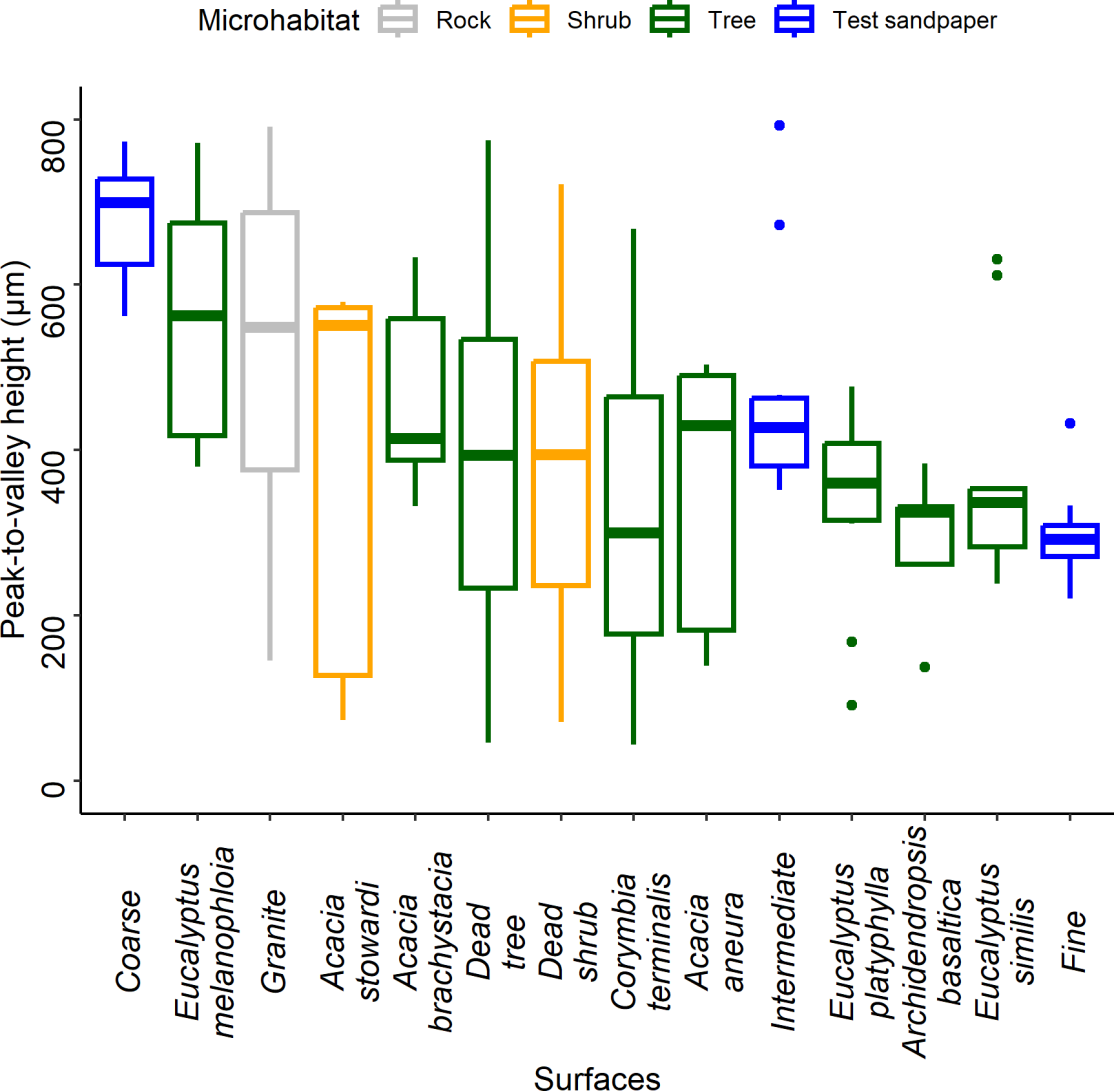
Peak-to-valley heights of natural microhabitats and test substrates. Colours represent habitat types used by diplodactylid geckos: trees (green), rocks (grey) and shrubs (orange). Test substrates (blue) correspond to the upper (coarse sandpaper), average (intermediate sandpaper) and lower (fine sandpaper) surface roughness of natural microhabitats.

We recorded the tree species and types of rock geckos used. For *O. castelnaui* (dead trees and *Eucalyptus melanophloia*), *O. coggeri* (granite) and *O. monilis* (*E. similis, E. platyphylla, E. melanophloia*, dead trees and granite), we used 10 measurements of peak-to-valley heights per substrate using a Landtek SRT-6223 surface profile gauge (accuracy: ± 5 μm; resolution: 0.1 μm/1 μm; range: 0–800 μm), and the mean of these values was used for further analysis [39,40]. For all other species (*O. cincta*—dead trees, *Acacia brachystacia* and *Archidendropsis basaltica; S. ciliaris*—dead trees, *Acacia stowardi* and *Corymbia terminalis; S. krisalys—Acacia aneura* and *A. brachystacia*), we obtained five peak-to-valley height measurements within 10 cm of the location where each individual was first sighted. We then assessed 16 commercially available sandpaper grits using the same methods and selected three that matched the upper, average and lower ranges of peak-to-valley heights recorded in the field. The chosen sandpapers—coarse (P40), intermediate (P60) and fine (P80) aluminium oxide (Active Abrasives Pty Ltd., Australia) were used in trials to test clinging ability (figure 1).

### (d) Function

We measured two functional traits: attachment (probability of holding onto a substrate versus falling) and clinging ability (shear force generation on inclines from which geckos never fell). Shear force (in Newtons) was measured using an ATI NANO 17-E force transducer (SI-50-0.5 Calibration, ATI Industrial Automation, USA) mounted beneath a three-dimensional-printed plastic test surface (18 × 8 cm). This surface was level, with an adjustable aluminium platform (47.5 × 25 cm) set at 45° or 90° inclines (electronic supplementary material, data S1). Surfaces were covered with the appropriate sandpaper grade for each trial. Each gecko was placed on the test surface by hand and allowed to take one step with each of its four feet, ensuring full engagement of its natural attachment system [39,40,48–50]. Once all four feet made contact, the gecko was pulled horizontally backwards using a 0.5 mm fishing line harness (Jarvis Walker Pty Ltd., Dandenong, Australia) attached to its inguinal region [39,40,50]. Individuals were tested in a randomized order with a 15 min rest between trials to prevent consecutive testing of the same gecko. One gecko was tested and then rested immediately, during which the next individual was tested. To eliminate bias caused by different observers, all trials were conducted by R.R.P. Gloves were always worn to prevent contamination of the geckos’ adhesive system.

Each individual was tested on all three sandpaper types at both 45° and 90° inclines, with three trials per substrate and orientation, totalling 18 trials per gecko. Shear force data were recorded for 5 s per trial, and each trial generated 3163 shear force measurements across three axes (*x*, *y* and *z*), automatically exported as a .csv file using ATI NI-DAQmx software (DAQ Express, v. 20.1, ATI Industries). As we focused on shear force in the primary direction of gecko attachment (distal-to-proximal plane), we extracted the maximum *x*-axis values as our measure of clinging performance. The transducer was zeroed before each trial. Attachment performance was recorded as a binomial variable: ‘attached’ (held on) or ‘failed to attach’ (fell off).

To assess the role of claws in attachment, all individuals were first tested with their claws intact within 5 days of shedding. Geckos exert higher shear force during this period, as their adhesive apparatus is rejuvenated through ecdysis [50]. Immediately after completing these trials, claw tips were clipped using microscissors, ensuring no damage to the underlying tissue [51] and minimizing potential harm to the setal fields. Following claw removal, geckos were allowed to rest until their next shedding event, enabling any potential damage to the setal fields, from testing or claw clipping, to be naturally repaired through ecdysis [50]. Once shedding was complete, trials were repeated within 5 days to assess attachment performance without claws. Experiments were conducted under James Cook University ethics permit A2691.

### (e) Statistical analysis

Attachment (the ability to hold on or not) was treated as a binary categorical response variable. To estimate the predicted probabilities of failure to attach on each surface, both with and without claws, we used a binomial logistic generalized linear mixed model in the glmmTMB package [52]. Explanatory variables included: claw presence/absence, the maximum shear force from three trials, gecko microhabitat of origin (arboreal, rock-dwelling, generalist), orientation (45° or 90°) and substrate type (coarse, P40 grit; intermediate, P60; fine, P80). As each individual was tested multiple times under different conditions, gecko ID was included as a random effect to account for intraspecific variation. Significant terms were identified using the Anova() function in the car package [53], and predictions were visualized using the ggemmeans() function in the emmeans package [54]. The relative contribution of claws was calculated by subtracting the probability of attachment with claws from the probability of attachment after claw removal.

To analyse the effect of claws on clinging ability (shear force generation), we compared two candidate models. In both models, shear force was the response variable. The first model included a three-way interaction between substrate, claw presence and microhabitat of origin. Since the impact of claw removal on clinging ability was expected to vary by substrate, the second model included an interaction between substrate and claw presence, with microhabitat of origin as an additive term. Because body size influences clinging ability such that larger animals exert greater force [26,31,55–57], we included mass as a fixed effect in both models. Individual gecko ID was included as a random factor to account for repeated measures. Shear force (in Newtons) was natural log-transformed after examining residuals. For these analyses, we used only data from inclined surfaces (45°), where geckos never failed to attach, as clinging ability could not be quantified when individuals fell (see §3). Models were compared using Akaike’s information criterion (AICc) via the AIC() function in the MuMIn package. Significant terms were identified using the Anova() function in the car package [54] with type III sum of squares. Post hoc tests were conducted using the emmeans package to compare the effect of claws on clinging ability among species within the same microhabitat and to compare clinging ability across species from different microhabitats. All analyses were performed in R using RStudio (v. 4.3.1) [58].

## 3. Results

### (a) Function—attachment

**Figure 2.**
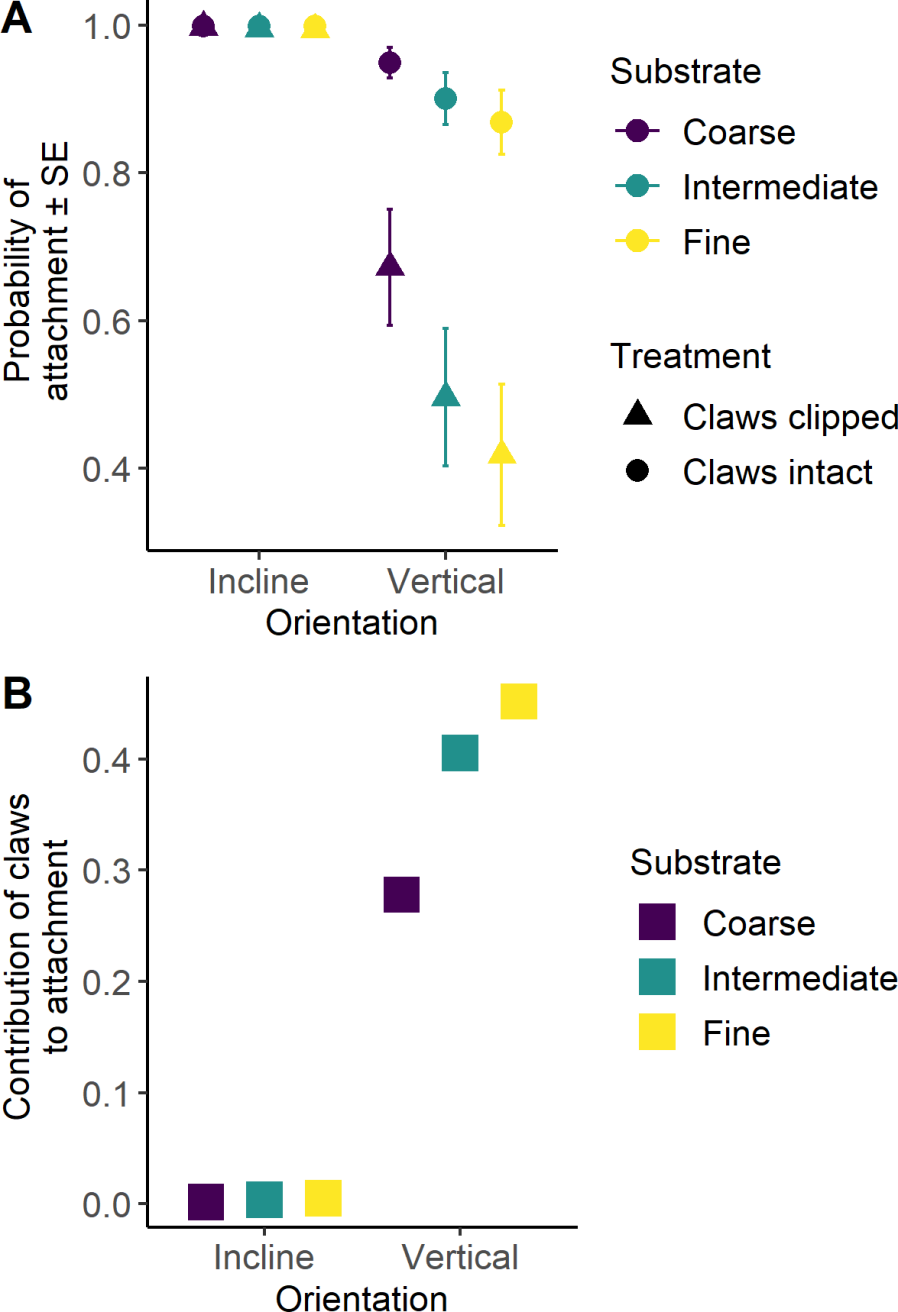
(A) Role of claws in attachment on inclines and vertical surfaces. Circles, attachment with claws intact; triangles, attachment without claws. (B) Relative contribution of claws to attachment in diplodactylid geckos. Purple symbols, coarse (P40-grit) sandpaper; green symbols, intermediate (P60-grit) sandpaper; yellow symbols, fine (P80-grit) sandpaper.

Claws were essential for attachment on vertical surfaces (90°), significantly increasing the probability of attachment. With claws intact on vertical surfaces, attachment probability was lowest on fine sandpaper (P80: 0.87 ± 0.38 s.e.), increased on intermediate sandpaper (P60: 0.90 ± 0.40 s.e.) and was highest on coarse sandpaper (P40: 0.95 ± 0.43 s.e.). On inclined surfaces (45°), attachment probability was higher across all sandpaper types (P40: 0.99 ± 1.16; P60: 0.99 ± 1.12; P80: 0.99 ± 1.10) compared with vertical surfaces (P40: 0.95 ± 0.43; P60: 0.90 ± 0.40; P80: 0.87 ± 0.38; figure 2A). In the absence of claws on vertical surfaces, attachment relied solely on adhesive pads, with the highest attachment observed on coarse surfaces (0.67 ± 0.36), followed by intermediate (0.50 ± 0.37) and fine-grained surfaces (0.42 ± 0.40; figure 2B). Without claws, the decline in attachment ability with decreasing surface roughness on vertical surfaces followed the same pattern observed with intact claws but was more pronounced. The probability of attachment was significantly lower on all vertical substrates compared with trials with claws (P40: *p* < 0.01, P60: *p* < 0.01, P80: *p* < 0.01; figure 2A).

Claw removal (*z* = 6.55, *p* < 0.01), shear force (*z* = −4.60, *p* < 0.01), orientation (*z* = 5.29, *p* < 0.01) and substrate type (P40: *z* = −5.15, *p* < 0.01; P60: *z* = 2.02, *p* < 0.05; P80: *z* = 2.78, *p* < 0.01) all significantly affected the probability of attachment (binary outcome: holding on versus falling off), according to the binomial logistic generalized linear mixed model. However, the microhabitat of origin had no significant effect on attachment probability.

At an incline (45°), most geckos attached successfully to all substrate types, regardless of claw presence (99% success on P40, P60 and P80). There was no significant difference in attachment probability among substrates or between geckos with and without claws at this angle (figure 2).

### (b) Function—clinging ability (shear force generation)

**Figure 3.**
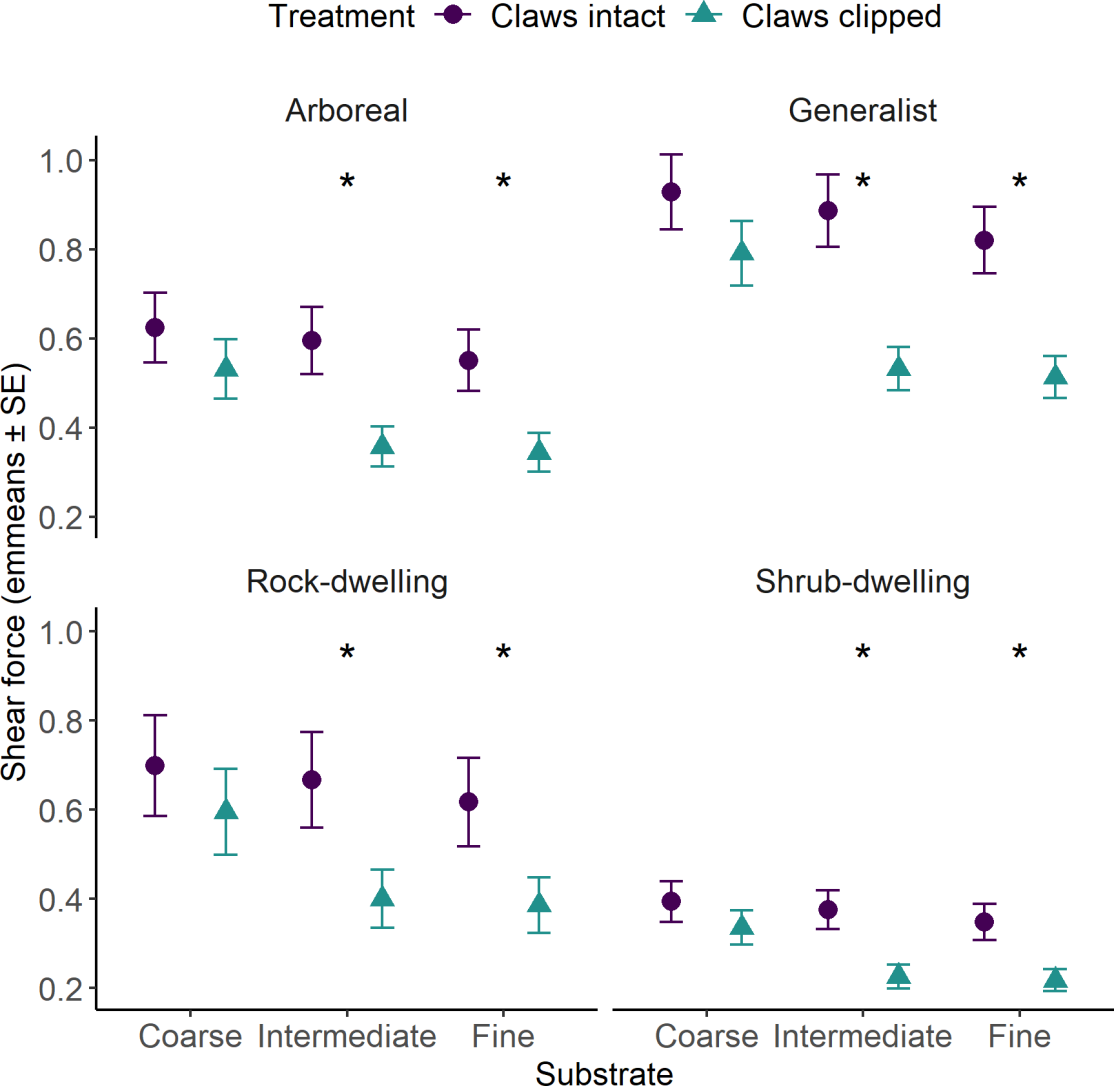
Role of claws in shear force on inclines (45°). Purple circles ± s.e., estimated marginal least square means (emmeans) of shear force exerted with claws; blue triangles ± s.e., emmeans of shear force exerted without claws. Asterisk * indicates significant differences between shear force with and without claws on the same substrate.

The most parsimonious model included the interaction between substrate type and claw presence, with microhabitat of origin as an additive term (AICc = 385.23, d.f. = 12). This model outperformed the alternative, which included a three-way interaction between substrate, claw presence and microhabitat of origin (AICc = 424.21, d.f. = 27). In the best-fitting model, there was a significant interaction between substrate type and claw presence on inclined surfaces (45°;* χ²* = 11.01, *p* < 0.01), indicating that the effect of claw removal on clinging ability varied among substrates. When claws were removed, clinging ability declined significantly on intermediate (P60) and fine-grained (P80) sandpapers (*p* < 0.01), with reductions of 0.24 N (P60) and 0.21 N (P80; estimated marginal least square means). However, clinging ability did not significantly change on coarse-grained (P40) surfaces (*p* = 0.51). These results mirror the attachment pattern, where adhesive pads alone were most effective on coarse surfaces, but their effectiveness declined as surface roughness decreased (figure 3).

The fixed effect microhabitat of origin was significant (*χ*² = 15.98, *p* < 0.01), indicating that species from different microhabitats exerted different levels of clinging ability. Generalist species generated greater clinging ability than shrub-dwelling geckos on all substrates (P40, P60 and P80), both with claws (*p* < 0.01) and without (*p* < 0.01). In contrast, arboreal and rock-dwelling species showed no significant difference in clinging ability, either with claws (*p* > 0.05) or without claws (*p* > 0.05), across all substrates. The magnitude of shear force generated with and without claws differed based on the microhabitats geckos used in nature. However, the intraspecific differences among substrates within each species followed the same trajectory. On coarse surfaces, shear force generation was not affected by claw removal, indicating that adhesive pads could function equally well on coarse surfaces in the absence of claws. On intermediate and fine surfaces, shear force generation was significantly greater with claws, indicating that claws were important to attachment on these surfaces while adhesive pads alone could not perform effectively (figure 3).

## 4. Discussion

### (a) Summary

We examined the attachment system of diplodactylid geckos, studying two functional traits: attachment and clinging ability, using surfaces that reflected the range of roughness of natural microhabitats. We found that claws were essential for attachment on vertical surfaces. Without claws, pads adhered best to coarse surfaces, and attachment declined linearly with decreasing surface roughness. Although attachment was lowest on fine surfaces, this is where claws contributed the most to attachment. For clinging ability, claws played an additive role on all inclined surfaces. By testing surfaces with roughness based on natural microhabitats, we demonstrated that pad performance trajectories and claw effectiveness in diplodactylid geckos vary with surface roughness, and that both pads and claws worked best on coarse surfaces.

### (b) Unexpected additive roles of claws and adhesive pads

Knowledge about roughness-dependent attachment in vertebrates is limited to only a few studies on lizards (e.g. [17,18,50,58,59]), with the majority of research focusing on invertebrates (e.g. stick insects [22–24]; cockroaches [21]). Our findings reveal unconventional adhesion patterns in diplodactylid geckos, where attachment and clinging ability did not decline with increasing surface roughness, regardless of claw presence. Compared with geckos, invertebrates possess attachment systems that are functionally more diverse and context-dependent, varying with ‘smooth’ or ‘hairy’ pad morphologies and involving both active and passive mechanisms [60,61]. Despite this complexity, hairy pads in insects also struggle on rough surfaces, with studies reporting minimal attachment [60,62]. For both smooth and hairy pad types, performance is typically highest on smooth substrates [63]. In contrast, diplodactylid geckos showed a decrease in both attachment and shear force as surface roughness declined. Especially in the absence of claws, their pads performed best on coarse surfaces, with effectiveness decreasing on intermediate- and fine-grained substrates—where other taxa typically perform better.

### (c) Dual functionality of diplodactylid adhesive pads on rough substrates

Why do diplodactylid geckos cling so effectively to rough surfaces, while the pads of other taxa perform poorly on similar substrates [17,18]? Our findings suggest that this ability is not solely due to claws, but rather a combination of claws and pads that enhance clinging on rough surfaces. The length and spacing of leaf-like lamellae on the pads of diplodactylid geckos (as opposed to basal pads) might better conform to the wavelength and amplitude of the rough surfaces tested in this study [64], as we purposely selected surfaces with a roughness similar to those they encounter in nature. In natural environments, the ideal conditions for maximizing surface contact of either pads or claws are probably rare; thus, the system may be overbuilt as a safeguard in suboptimal conditions [65]. Studies have shown that adhesive pads alone can generate shear forces several times greater than a gecko’s body mass. Our findings of the additive function suggest that adhesive pads may serve as a backup mechanism when claws become blunt or damaged. Furthermore, a small proportion of pad-bearing lineages have partially or completely reduced their claws, indicating that under certain evolutionary scenarios, claws can become completely obsolete [33], providing increased evidence for pads being self-sufficient in some instances.

### (d) Functional and ecological implications

Diplodactylid geckos could attach well and generate shear force on coarse surfaces in the absence of claws. Claws are prone to damage and wear and tear due to constant interaction with the environment [66,67]. Therefore, it is important that attachment occurs even under these suboptimal scenarios, as effective movement is crucial for predator avoidance, prey capture and mate acquisition [68–70]. The ability of pads to function on rough surfaces, even without claws, allows geckos to exploit microhabitats where claws alone would typically dominate. Additionally, the ability to attach and generate shear force without claws may provide advantages by providing more effective attachment, which could impact fitness and survival [69,71–73].

We did not find a direct relationship between attachment capabilities and microhabitat use, but our results showed that on coarse surfaces, pads play a greater role in attachment, while being less effective on intermediate and fine surfaces. Does this indicate that pads provide backup on coarse surfaces that are conventionally considered to be more challenging for such attachment systems? Our observations of microhabitat use show that diplodactylid geckos rarely use very smooth surfaces, such as leaves, in nature, instead favouring microhabitats comprising coarse substrates like bark and branches. Pillai *et al.* [40] showed that some diplodactylids preferentially chose coarse surfaces as microhabitats under laboratory conditions and also performed better on these substrates, supporting this notion. Our findings are consistent with these observations and thus support the idea that the attachment system of diplodactylids could potentially be better suited to coarse microhabitats in nature. Furthermore, it is unknown whether species with smaller claws attach more effectively to fine-grained microhabitats, while species with larger claws favour rougher environments [74]. Future studies should quantify the morphology of these structures to better understand their role in microhabitat choice.

In our study, species that used a range of microhabitats (scansorial generalists) in nature exerted greater shear force across all substrates, with and without claws, compared with species that used only one microhabitat (specialists), consistent with [40]. Generalist species are often expected to have lower functional specialization while maintaining adaptability across diverse microhabitats (the ‘jack of all trades, master of none’ concept) [75]. However, trade-offs of this nature do not always occur [76], and here we observed no trade-off. Possibly, generalist species need greater functionality to meet multiple challenges in the form of surfaces that are subpar for ideal attachment and contact. Future research should further investigate the fine-scale structure of microhabitats used by diplodactylid geckos to understand these functional adaptations.

Preliminary observations of the setal fields in diplodactylid geckos reveal distinct morphological patterns that may help explain differences in adhesive performance (personal observation). Generalist species appear to have longer and denser setae than specialist arboreal, rock-dwelling and shrub-dwelling species. Long, dense setae probably contribute to their ability to generate greater shear force, even without the aid of claws. In contrast, shrub-dwelling *Strophurus* species had shorter, sparser setae, which may account for their comparatively lower adhesive performance. These results suggest unconventional patterns of performance among diplodactylids that may be driven by variation in the structure of the adhesive system. Our findings offer a foundation for future research into the morphology of setal fields and claws, and how their interaction with natural surfaces contributes to shear force generation.

### (e) Broader connections to biomimetics

Attachment to coarse surfaces has historically been a challenge in developing biomimetics; therefore, identifying a clade capable of effectively clinging to ‘difficult’ substrates could inspire innovations that expand biomimetic applications across a wider range of surfaces. Additionally, examining the structural characteristics of microhabitats used by these geckos enhances our understanding of their largely understudied ecology. Understanding how adhesive toepads function in nature not only deepens our knowledge of the relationship between ecology and biomechanics (ecomechanics) but also informs biomimetic design [38,77,78].

## 5. Conclusion and future studies

Research on organismal attachment has traditionally focused on the function and biomimetic potential of attachment systems [38,61,79], while evolutionary and ecological perspectives have only recently received increased attention (e.g. [31,40,77,79,80]). Our findings demonstrate the distinct functional roles of claws and adhesive pads in diplodactylid geckos, contributing to a broader understanding of how organisms attach to natural surfaces. This work helps bridge the gap between functional morphology and ecological context. It also provides a framework for future investigations into the morphology of claws and pads, particularly their structural integration or division of labour in enabling attachment on rough, irregular surfaces. Additionally, we emphasize that diplodactylid geckos, an often-understudied group, represent a valuable model system for advancing research at the intersection of morphology, ecology and functional performance.

## Data Availability

Data are publicly available at [81]. Supplementary material is available online [82].
